# Reply: Epidemiological cut-off values for a 96-well broth microdilution plate for high-throughput research antibiotic susceptibility testing of *M. tuberculosis*

**DOI:** 10.1183/13993003.00426-2023

**Published:** 2023-05-04

**Authors:** The CRyPTIC Consortium

**Affiliations:** For a list of all members of the CRyPTIC Consortium and their affiliations, please see the section at the end of the original manuscript

## Abstract

Herein we respond to the letters from G. Kahlmeter and J. Turnidge and from C.U. Köser and F.P. Maurer together, as there is an overlap in their scope. Both letters to the editor addressed our publication titled “Epidemiological cut-off values for a 96-well broth microdilution plate for high-throughput research antibiotic susceptibility testing of *M. tuberculosis*” [1].

## Abstract 2

**The recent publication by the @crypticproject proposing ECOFF/ECVs for research attracted two letters to the editor. Here is the response from the CRyPTIC Consortium to both.** https://bit.ly/40vRLye

*Reply to G. Kahlmeter and J. Turnidge, and C.U. Köser and F.P. Maurer*:

Herein we respond to the letters from G. Kahlmeter and J. Turnidge and from C.U. Köser and F.P. Maurer together, as there is an overlap in their scope. Both letters to the editor addressed our publication titled “Epidemiological cut-off values for a 96-well broth microdilution plate for high-throughput research antibiotic susceptibility testing of *M. tuberculosis*” [1].

G. Kahlmeter and J. Turnidge are leading officers of the European Committee on Antimicrobial Susceptibility Testing (EUCAST) and their letter provides an excellent and clear exposition of their views about deriving epidemiological cut-off values (ECOFFs) to meet the rigors of guidelines to interpret antimicrobial resistance provided by a highly recognised scientific committee, of which they are prominent members. The Comprehensive Resistance Prediction for Tuberculosis: an International Consortium (CRyPTIC), in contrast, is a research consortium. It consists of over 20 participating countries and includes leading mycobacteriologists as co-investigators, some of whom are active in EUCAST and other standards organisations. The planning and design of the project was undertaken in late 2015 and early 2016, before relevant specific guidance was published for *Mycobacterium tuberculosis* broth microdilution (BMD) testing or ECOFF determination. We aimed to discover the worldwide genomic variation of *M. tuberculosis* conferring resistance to each of 13 drugs. This needed a clear metric distinguishing susceptible from resistant strains and the ECOFF/ECV statistic was ideal for this purpose (discussed below). Robust minimum inhibitory concentration (MIC) measurement of >20 000 samples, oversampled for resistance, was needed to achieve sufficient statistical power to measure MICs associated with less common variants. Considerable attention was given to assuring that high quality scientific standards were met, thus providing robustness for the results of the study (discussed below). Crucially, at the initiation of the study, no guidance for MIC measurements or determination of ECOFFs/ECVs for *M. tuberculosis* was published, including by EUCAST or the Clinical and Laboratory Standards Institute (CLSI), respectively.

As a research group, the CRyPTIC Consortium considers EUCAST, CLSI and the World Health Organization (WHO) to be key organisations who provide drug testing guidance to ensure that solid standards are met for regulatory certification and patient safety.

G. Kahlmeter and J. Turnidge state the publication has numerous misconceptions, to which we respond as follows:

1) They imply that we attempted to alter the standard procedures for determining clinical breakpoints, ECOFFs and how to determine quality control (QC) of MIC testing. We disagree: the paper did not give guidance on how to address these issues. We agree that guidance is clearly a matter for the standards agencies. We simply provided, as is usual for all research papers, the detailed methodology that we used.
2) The authors of the CRyPTIC Consortium fully recognise and respect that the standards agencies operate within strict guidelines. Generating a research ECOFF/ECV statistic from our data provided a convenient method to characterise MICs within our study. At the time no relevant guidance for tuberculosis (TB) broth microtitre dilution had been published. In our study, 14 respected laboratories (more than the three or five needed in guidance) across the world (some being WHO TB Supranational Laboratories) generated data controlled with H37Rv, following the instructions for use of the MYCOTBI AST Plate (which is in line with much of the QC needed for BMD assays). Each laboratory contributed many more than 200 isolates, and some in the thousands.
3) The approach we have taken was for research and, this is not only explicitly specified in the title of the paper, but also repeatedly referred to in the body of the paper and is clearly addressed in the first paragraph of the discussion. The findings are clearly not to be used for clinical management of patients – such guidance is the role for standards agencies. We await the publication of ECOFF/ECVs by the standards agencies for all the drugs used for treating TB. This will provide an opportunity to formally quantify the extent of any divergence of the results we report from a reference standard.

We would like to thank C.U. Köser and F.P. Maurer for their interest in the data collected by the CRyPTIC Consortium project [1]. We are aware there are several shortcomings and agree with the assertion by C.U. Köser and F.P. Maurer that our data have several limitations.

*Choice of MIC assay and QC*. We agree that there is a clear global need for a high-quality BMD assay for quantitative, inexpensive antimicrobial susceptibility testing (AST) of *M. tuberculosis* complex (MBTC). Care was taken to choose a research-only microtitre AST plate which incorporated 13 drugs and was based on the now CE-marked MYCOTBI AST Plate (Thermo Fisher Scientific Inc., Waltham, MA, USA) which has both a robust manufacturing process and clear instructions for use.

The UKMYC BMD plate was developed for research use by the CRyPTIC Consortium (in collaboration with Thermo Fisher) and, as described above, was used to assay over 20 000 samples [2]. Over 15 000 of these samples also underwent whole genome sequencing. We hope that the UKMYC plates and the CRyPTIC dataset stimulate discussion in the community, including by the relevant national and international bodies, about the design and adoption of a BMD plate for TB AST.

The authors are correct that minimising intra- and inter-laboratory technical variability is key when developing a BMD plate and accurate measurement was also essential for CRyPTIC's research goals. All MICs were therefore measured using three independent methods: by the laboratory scientist, by some computer software [3] and by a citizen science project, BashTheBug [4]. By taking the consensus, CRyPTIC was able to reduce the component of technical variation arising from subjectivity when measuring a BMD plate.

Regular monitoring using one or more QC strains is also important. Whilst the CRyPTIC standard operating procedure specified that participating laboratories run QC checks using the H37Rv strain, it did not require the laboratories upload the control MICs to our centralised database, hence some laboratories appeared not to contribute to the QC, if taking histograms in figure S2 at face value. CRyPTIC was first and foremost a research project and the participating laboratories collected vastly different numbers of samples at differing rates, but following their internal quality measures. The research accepted the programme would be undertaken without an external quality assurance system (none existed) for TB BMD plates, thus, in this respect, it could not fully conform to ISO 15189 as would be typical for a routine public health laboratory test.

*Range of dilutions on plate and truncated distributions*. The choice of dilutions for each drug presented a challenge in the design of the research plates. We were interested in associating the sample genotype with the change in MIC and hence were equally interested in low and high MICs. Thus, for CRyPTIC to have used a plate design that ensured untruncated distributions would have required at least two distinct plate designs for the 13 antibiotics, increasing cost and complexity and thereby reducing the number of samples collected and the statistical power of the study. We collectively took the decision that perfection is the enemy of the good and decided, given our limited resources and research aims, to characterise more samples. In addition, during the study we modified the plate design to further explore the truncated distributions.

*Genomically defined* versus *phenotypically defined wildtype*. While G. Kahlmeter and J. Turnidge refute the idea that a genetically defined wildtype is useful, there is a growing explicit dependence on identifying mechanisms of resistance for classifying wildtype and non-wildtype status of TB rather than relying on inference from phenotypic MIC distributions. Using genomic data to confidently confirm a wildtype population is gaining credibility [5, 6]. The use of phenotypically defined wildtypes (pWT) relies on the implicit assumption that the phenotypically non-wildtype (pNWT) MIC distribution(s) are sufficiently distinct from that of phenotypically wildtype samples. This does not always hold, even for drugs accepted to conform to the above assumption: the small increase in isoniazid MIC introduced by *fabG1* promoter mutations (figure 6a) cannot be discerned unless one has genetic information. For other drugs, such as ethambutol, the measured MIC distribution is the convolution of several underlying smaller MIC distributions, obfuscating the pNWT. Whilst for other drugs, there are many mutations peri-ECOFF/ECV (“disputed mutations”) which each measurably elevate the point estimate MIC and are only identifiable using genetics [7]. To complicate matters further, we have identified a strong lineage effect on the MIC for some of the drugs. These effects are only evident when one has genetic information for the samples.

We elected to analyse our data stratified by genomic variation to determine the MIC distribution for each distinct variant and to explore the possibility of the existence of major genetic interactions. In addition, as C.U. Köser and F.P. Maurer argue, using genomically defined wildtypes minimises the risk of overestimating the ECOFF in cases where the genes determining resistance are known and the MIC distribution is truncated. The available data can also be easily re-analysed whenever a new resistance-determining gene is discovered.

Clearly, the field is moving towards genomics-based prediction of antituberculosis resistance in TB as it is faster, cheaper, more precise and less variable than phenotyping.

*Statistical methodology (interval regression* versus *ECOFFinder)*. The conclusions of C.U. Köser and F.P. Maurer appear to rest, in part at least, on the use of ECOFFinder [8] to estimate the ECOFF. ECOFFinder, which is the only statistical method recommended by EUCAST and one of several methods recommended by the CLSI [6], relies on a heuristic that assumes the MIC distribution is discrete, which it is not, and encompasses the entire lower range of observed MICs to avoid truncation. ECOFFinder also assumes that the pWT and pNWT are sufficiently distinct and separated that they identify the presence of the latter on the fit of a log-normal to the former, enabling numerical detection. As rehearsed above, there are many examples in the CRyPTIC dataset where these assumptions are not true. The ECOFF/ECV is simply defined by EUCAST and others as the MIC below which a stated percentile (often 99%) of phenotypically wildtype samples lie and hence if one has, at most, several hundred samples as is typical, fitting a log-normal distribution is an expedient but not a required step.

Instead, the correct statistical approach when fitting MIC data is interval regression [9, 10], as this correctly represents each MIC as having a lower and upper bound, rather than being a discrete datapoint, and does not depend on all assays having identical boundaries, thus allowing results collected using different plate designs to be merged. Interval regression deals with truncated data (lower bound unknown) by returning estimates with wider confidence intervals. With extreme truncation, when the mode is not discernible, the estimates will become less reliable. In our data this was seen for three of the 13 antibiotics (amikacin, rifabutin, delamanid) on the UKMYC6 BMD plate (figure 2).

The results of these two methods were compared in the paper. Fortuitously, as has been recently specified by the CLSI [6], this approach is consistent with their statement that: “the sponsor should explain and justify the methodology that is used”.

We are delighted that the authors believe the CRyPTIC data should have been included in the international MIC distribution database to analyse together with other MIC data. All the data are freely available [11] and our results are also reproducible (in a web browser) [12]. We welcome researchers to analyse the CRyPTIC data in more detail and potentially provide alternative interpretations that can then be compared to our published approach. Stepping back, we hope that CRyPTIC, and other similar projects, are the start of clinical microbiology joining other sciences in the realm of big data; however, this will require changes to how we analyse, store and approach data which will no doubt promote further necessary discussion and debate.

## Shareable PDF

10.1183/13993003.00426-2023.Shareable1 This one-page PDF can be shared freely online.
Shareable PDF ERJ-00426-2023.Shareable
