# Supplementary material for: Reply: Epidemiological cut-off values for a 96-well broth microdilution plate for high-throughput research antibiotic susceptibility testing of M. tuberculosis
Source: Eur Respir J. 2023 May 4;61(5):2300426. doi: 10.1183/13993003.00426-2023 (PMC10160797; doi:10.1183/13993003.00426-2023)
Supplement: Supplementary file 1 [file ERJ-00426-2023.Shareable.pdf]

SHAREABLE PDF

Reply: Epidemiological cut-off values for a 96-well broth microdilution plate for high-throughput research antibiotic susceptibility testing of *M. tuberculosis*

The CRyPTIC Consortium

For a list of all members of the CRyPTIC Consortium and their affiliations, please see the section at the end of the original manuscript.

Corresponding author: Philip W. Fowler (philip.fowler@ndm.ox.ac.uk)

Check for updates

Shareable abstract (@ERSpublications)
The recent publication by the @crypticproject proposing ECOFF/ECVs for research attracted two letters to the editor. Here is the response from the CRyPTIC Consortium to both.
https://bit.ly/40vRLye

Cite this article as: . Reply: Epidemiological cut-off values for a 96-well broth microdilution plate for high-throughput research antibiotic susceptibility testing of *M. tuberculosis*. *Eur Respir J* 2023; 61: 2300426 [DOI: 10.1183/13993003.00426-2023].

This single-page version can be shared freely online.

Copyright ©The authors 2023.

This version is distributed under the terms of the Creative Commons Attribution Licence 4.0.

Received: 10 March 2023
Accepted: 15 March 2023

Reply to G. Kahlmeter and J. Turnidge, and C.U. Köser and F.P. Maurer:

Herein we respond to the letters from G. Kahlmeter and J. Turnidge and from C.U. Köser and F.P. Maurer together, as there is an overlap in their scope. Both letters to the editor addressed our publication titled “Epidemiological cut-off values for a 96-well broth microdilution plate for high-throughput research antibiotic susceptibility testing of *M. tuberculosis*” [1].
